# Glucose Activates Lysine-Specific Demethylase 1 through the KEAP1/p62 Pathway

**DOI:** 10.3390/antiox10121898

**Published:** 2021-11-26

**Authors:** Chiao-Yun Lin, Chen-Bin Chang, Ren-Chin Wu, Angel Chao, Yun-Shien Lee, Chi-Neu Tsai, Chih-Hao Chen, Chih-Feng Yen, Chia-Lung Tsai

**Affiliations:** 1 Gynecologic Cancer Research Center, Linkou Chang Gung Memorial Hospital, Taoyuan 333, Taiwan; chiaoyun@cgmh.org.tw (C.-Y.L.); b9505039@cgmh.org.tw (C.-B.C.); angel945@cgmh.org.tw (A.C.); 2 Department of Obstetrics and Gynecology, Linkou Chang Gung Memorial Hospital and Chang Gung University College of Medicine, Taoyuan 333, Taiwan; yen2158@cgmh.org.tw; 3 Department of Pathology, Linkou Chang Gung Memorial Hospital and Chang Gung University College of Medicine, Taoyuan 333, Taiwan; qby@cgmh.org.tw; 4 Department of Biotechnology, Ming-Chuan University, Taoyuan 333, Taiwan; bojack@mail.mcu.edu.tw; 5 Department of Surgery, Graduate Institute of Clinical Medical Sciences, Chang-Gung University, New Taipei Municipal Tucheng Hospital, New Taipei City 236, Taiwan; pink7@cgu.edu.tw; 6 Department of Plastic and Reconstructive Surgery, Chang Gung Memorial Hospital at Keelung, Chang Gung University College of Medicine, Taoyuan 333, Taiwan; cjh5027@cgmh.org.tw; 7 Genomic Medicine Research Core Laboratory, Chang Gung Memorial Hospital, Taoyuan 333, Taiwan

**Keywords:** p62, KEAP1, LSD1, NRF2, endometrial cells

## Abstract

Endometrial cancer incidence increases annually. Several risk factors, including high glucose intake, are associated with endometrial cancer. We investigated whether glucose affects lysine-specific demethylase 1 (LSD1) expression and the responsible molecular mechanisms. A high concentration of glucose stimulated p62 phosphorylation and increased LSD1 protein expression. Knockdown of p62 or treatment with mammalian target of rapamycin (mTOR), transforming growth factor-β activated kinase 1 (TAK1), casein kinase 1 (CK1), and protein kinase C (PKC) inhibitors abrogated glucose-regulated LSD1 expression. Unphosphorylated p62 and LSD1 formed a complex with Kelch-like ECH-associated protein 1 (KEAP1) and were degraded by the KEAP1-dependent proteasome. Phosphorylated p62 increased LSD1 protein expression by escaping the KEAP1 proteasome complex. LSD1 and KEAP1 interaction was enhanced in the presence of the nuclear factor erythroid 2-related factor 2 (NRF2) protein. LSD1 also participated in antioxidant gene regulation with NRF2. In diabetic mice, increasing LSD1and phospho-p62 expression was observed in uterine epithelial cells. Our results indicate that glucose induces p62 phosphorylation through mTOR, TAK1, CK1, and PKC kinases. Subsequently, phospho-p62 competitively interacts with KEAP1 and releases NRF2–LSD1 from the KEAP1 proteasome complex. Our findings may have public health implications for the prevention of endometrial cancer.

## 1. Introduction

Endometrial cancer (EC) originates from epithelial layer of uterus cavity, and its incidence and mortality increased between 1990 to 2017 [1]. Most patients with endometrial cancer are postmenopausal women, and only about 14% of patients are 40 years of age or younger [2]. The main risk factor is endogenous estrogen or estrogen-like drugs used in hormone therapy, such as tamoxifen, which cause endometrial cell pathology [3].

In addition to estrogen, some metabolic abnormalities such as obesity-associated hyperglycemia [4], hypertension [5], and hyperlipidemia [6] are considered risk factors of endometrial cancer. Patients with high blood sugar have an increased risk of developing breast cancer, hepatocellular carcinoma, pancreatic cancer, colorectal cancer, bladder cancer, and endometrial cancer [7]. A study indicated that the odds ratio of endometrial cancer with metabolic-related symptoms was 1.62 to 3.83, of which the odds ratio of type 2 diabetes was 2.18 [6]. High blood sugar can induce cancer cells to produce energy through glycolysis and the tricarboxylic acid (TCA) cycle to support the growth and proliferation of cancer cells [8,9,10], migration [11,12], chronic inflammatory cytokines to promote tumor-favorable microenvironment [13]. In addition, high glucose can induce genomic instability and gene mutation in cells [14]. While much evidence supports that glucose promotes the progression of cancer, the mechanisms still require in-depth study [15].

Glycolysis is a major pathway that entails the conversion of glucose to produce amino acids, nucleotides, and lipids to support cancer cells’ high proliferation rate and survival [16]. However, it also generates reactive oxygen species (ROS) that can induce cell death. To eliminate elevated ROS in cancer cells, several antioxidant mechanisms, such as the Kelch-like ECH-associated protein 1–nuclear factor erythroid 2-related factor 2 (KEAP1–NRF2) pathway, are activated [17]. NRF2 is a cap’n’collar basic region leucine zipper transcription factor [18] and activates downstream antioxidant genes by binding to antioxidant-responsive elements [19]. Under normal conditions, NRF2 remains at a low expression level by forming a complex with KEAP1 that can be degraded through the ubiquitin-proteasome system [17]. In the canonical pathway, increased oxidative stress can modify cysteine residues of KEAP1 and subsequently stabilize NRF2 by blocking the KEAP1–NRF2 interaction [20]. Moreover, ROS can induce the phosphorylation of serine 349 (S349) in p62 to complete the KEAP1–NRF2 interaction and stabilize NRF2 [21,22].

Lysine-specific demethylase 1 (LSD1) is a histone demethylase that removes the methyl group from histone H3 lysine 4 (H3K4) and H3 lysine 9 through oxidation [23]. LSD1 is overexpressed in many cancers, including EC [24], and promotes tumorigenesis [25]. Apart from the regulation of LSD1, protein stability also plays a vital role in cancer cells. Phosphorylation by GSK3β [26,27] or methylation [28,29] of the LSD1 protein can cause deubiquitination and stabilize the LSD1 protein in cancer cells. Moreover, a high concentration of glucose can induce LSD1 expression in retinal endothelial cells and the retina of diabetic rats [30], but the mechanistic details of how LSD1 is induced by glucose are generally unclear.

This study examined the molecular mechanisms by which glucose induces LSD1 expression and subsequent regulation of NRF2 downstream target genes. We determined that LSD1 forms a complex with KEAP1, p62, and NRF2, and p62 maintains LSD1 stability through a competitive interaction with KEAP1, which prevents KEAP1-mediated degradation. Moreover, the LSD1–KEAP1 interaction depends on NRF2 expression. Activated NRF2–LSD1 can regulate downstream antioxidant genes. We also demonstrated differential expression of LSD1, NRF2, phospho-p62, and KEAP1 in streptozotocin (STZ)-induced diabetic female mice. These findings may suggest a connection between glucose intake and gene expression in EC.

## 2. Materials and Methods

### 2.1. Cell Culture and Treatment of Cell Lines

HEK293 and HEC1B cells were obtained from the American Type Culture Collection (Manassas, VA, USA), and Ishikawa cells (human EC cells) were provided by Dr. Masato Nishida (Kasumigaura Medical Center, Ibaraki, Japan) [31]. ARK2 cells were gifts from Dr. Alessandro D. Santin (Yale University School of Medicine, New Haven, CT, USA). The HEK293 cells were cultured in DMEM/F12 with 10% fetal bovine serum and 1% penicillin and streptomycin. The Ishikawa cells were maintained in an alpha-MEM medium with 15% fetal bovine serum and 1% penicillin and streptomycin. The HEC1B and ARK2 cells were cultured in RPMI with 10% fetal bovine serum and 1% penicillin and streptomycin. The cells were maintained in 5.5, 11 or 22 mM glucose RPMI with 10% FBS for 48 h to perform indicated experiments. The following drugs were used in this study: a proteasome inhibitor (MG132; Sigma-Aldrich, St. Louis, MO, USA; concentration: 5 μM), mammalian target of rapamycin (mTOR) inhibitor that activates autophagy (everolimus; Novartis Pharma AG, Basel, Switzerland; concentration: 1 μM), protein kinase C (PKC) inhibitor (sotrastaurin; MedChemExpress, Monmouth Junction, NJ, USA; concentration: 1 μM), transforming growth factor-β activated kinase 1 (TAK1) inhibitor (takinib; MedChemExpress, Monmouth Junction, NJ, USA; concentration: 1 μM), casein kinase inhibitor (PF-670462; MedChemExpress, Monmouth Junction, NJ, USA; concentration: 1 μM), and glucose (50% glucose, Taiwan Biotech, Taoyuan, Taiwan).

### 2.2. Tissue Specimens

This study was reviewed and approved by the Institutional Review Board of Linkou Chang Gung Memorial Hospital (approval number: 201801952B0A3). Signed informed consent was obtained before surgery and tissue collection. Endometrial tissues were obtained from surgical specimens of fertile women with regular menstrual cycles who received laparoscopy or hysterectomy for benign gynecologic conditions.

### 2.3. Isolation and Maintenance of Primary Endometrial Stroma Cells

The minced surgical endometrial tissues were digested in Hanks’ Balanced Salt Solution (Invitrogen, Waltham, MA, USA) with collagenase B (15 U/mL; Roche, Basel, Switzerland), deoxyribonuclease I (150 U/mL, Roche, Basel, Switzerland), and penicillin/streptomycin at 37 °C for 60 min under agitation. Primary endometrial stroma cells were isolated through passage through 40-μm cell sieves (BD, Franklin Lakes, NJ, USA) and were cultured in DMEM/F12 with 10% fetal bovine serum [32]. 

### 2.4. DNA Construction

Full-length and truncated KEAP1 and NRF2 constructs were amplified from cDNA vectors (Sino Biological, Beijing, China) and ligated into the pNTAP expression vector (Agilent Technologies, Santa Clara, CA, USA) by using an In-Fusion HD cloning kit (Clontech, Mountain View, CA, USA). FLAG–LSD1 and HA-p62 constructs have been described [27,33]. HA-p62 S349D and HA-p62 S349A were constructed using overlap extension polymerase chain reaction (PCR), for which a Q5 site-directed mutagenesis kit (New England Biolabs, Ipswich, MA, USA) was employed. All primers used to generate DNA expression vectors are reported in Supplementary Table S1.

### 2.5. DNA and siRNA Transfection

For DNA transfection, HEK293 cells and the GenJet reagent (SignaGen Laboratories, Frederick, MD, USA) were used at a ratio of 1µg DNA to 2 µL GenJet in DMEM/F12 with 10% FBS. The transfection of the Ishikawa cells took 48 h and was performed using Lipofectamine 2000 (Invitrogen, Waltham, MA, USA) according to the manufacturer’s instructions. For siRNA transfection, Ishikawa and HEC1B cells were transfected through p62 and KEAP1 siRNA (Santa Cruz Biotechnology, Dallas, TX, USA) by using the Lipofectamine RNAiMAX reagent (Invitrogen, Waltham, MA, USA) for 72 h, and silencing efficacy was confirmed through Western blot.

### 2.6. RNA Extraction and Real-Time Q-PCR

Total RNA was isolated with the TOOLSmart RNA Extractor (Toolsbiotech, New Taipei City, Taiwan). First-strand cDNA for RT-QPCR was synthesized with an oligo-T primer by using the HiScript I first-strand cDNA synthesis kit (Bionovas, Toronto, Canada). The primers used in the SYBR green gene expression assay are displayed in Supplementary Table S1.

### 2.7. Cell Proliferation Assay

After siRNA was transfected for 48 h in a 96-well plate (bromodeoxyuridine [BrdU] assay) or in a six-well plate with a glass cover (Ki 67 staining), cells were treated with various concentrations of glucose for another 24 h. DNA synthesis activity was measured with a BrdU ELISA kit (Roche, Basel, Switzerland), after having added BrdU for 2 h. The expression levels of the endogenous proliferation marker-Ki67 (cell signaling, Danvers, MA, USA) were detected through immunostaining [34].

### 2.8. Western Blot 

After lysing the cells in a RIPA buffer (150 mM NaCl, 20 mM Tris-Cl pH 7.5, 1% Triton X-100, 1% NP40, 0.1% SDS, and 0.5% deoxycholate) in the presence of proteinase and phosphatase inhibitors (Bionovas, Toronto, ON, Canada), the proteins in the cell lysates were separated through sodium dodecyl sulphate–polyacrylamide gel electrophoresis (SDS-PAGE) and subsequently transferred onto nitrocellulose membranes [35]. The following antibodies were used: KEAP1 (Abclonal, Woburn, MA, USA), p62 (GeneTex, Hsinchu, Taiwan), phosphorylated (Ser349) p62 (Cell Signaling Technology, Danvers, MA, USA), FLAG tag (Sigma-Aldrich), CBP tag (calmodulin-binding peptide, Santa Cruz, for pNTAP expression vector), ubiquitin (Cell Signaling Technology, Danvers, MA, USA), LSD1 (Cell Signaling Technology, Danvers, MA, USA), Mono-Methyl-Histone H3 (Lys4; H3K4me1, Cell Signaling Technology, Danvers, MA, USA), and GAPDH (Santa Cruz Biotechnology, Dallas, TX, USA). The corresponding horseradish peroxidase–conjugated antibodies were obtained from Santa Cruz Biotechnology, and chemiluminescence reagents were acquired from Millipore (Burlington, MA, USA). The signal intensity of autoradiograms was quantified with ImageJ software after normalization to the corresponding GAPDH intensity.

### 2.9. Immunoprecipitation

Cells were lysed in a cell lysis buffer (20 mM Tris-Cl pH7.4, 25 mM NaCl, and 0.1% NP40) containing proteinase inhibitors and subsequently subjected to an overnight incubation with streptavidin beads (Invitrogen, Waltham, MA, USA) used for the pNTAP vector, M2 beads (Sigma-Aldrich, St. Louis, MO, USA) for the FLAG tag protein, or protein A beads for the anti-KEAP1 antibodies at 4 °C under agitation. After three washes with a buffer (20 mM Tris-Cl pH 7.4 and 25 mM NaCl), pulled-down complexes were subjected to SDS-PAGE and detected with specific antibodies. 

### 2.10. Confocal Microscopy

After seeding in a cover slip glass overnight, the Ishikawa cells were fixed with 4% paraformaldehyde, permeated with cold acetone for 20 min at −20 °C, and incubated in a blocking buffer (Thermo Fisher Scientific, Waltham, MA, USA) for 1 h at room temperature. Endogenous proteins were detected by incubating cells with antibodies against NRF2 (Cell Signaling, Danvers, MA, USA), LSD1 (Abnova, Taipei, Taiwan), and control immunoglobulin G (IgG; Santa Cruz Biotechnology, Dallas, TX, USA); this was followed by exposure to the corresponding fluorescent antibodies (Alex-fluor-488 or Alex-fluor-546; Invitrogen, Waltham, MA, USA). Finally, slides were examined with a Leica TCS SP2 confocal laser scanning microscope (Leica Microsystems GmbH, Wetzlar, Germany). 

### 2.11. Proximity Ligation Assay

Human endometrial specimens were deparaffinized and double-stained using the following antibodies: LSD1 (Abnova, Taipei, Taiwan), KEAP1 (Proteintech, Rosemont, IL, USA), NRF2 (GeneTex, Hsinchu, Taiwan), p62 (GeneTex, Hsinchu, Taiwan), and control IgG (Cell signaling, Danvers, MA, USA). Protein interactions were accomplished using the Duolink In Situ Starter Kit (Sigma-Aldrich, St. Louis, MO, USA) according to the manufacturer’s protocol. Slides were examined using a Leica TCS SP2 confocal laser scanning microscope.

### 2.12. Animal Experiments and Immunohistochemistry

All animal experiments were reviewed and approved by the Institutional Animal Care and Use Committee of Chang Gung Memorial Hospital (approval number: 2019061101). Diabetes was induced in 8-week-old female C57BL/6 mice by injecting them intraperitoneally with 40 mg/kg STZ (Sigma-Aldrich, St. Louis, MO, USA) within the first five days of the experiment. One month later, diabetes was confirmed through blood glucose concentrations of more than 250 mg/dL. Twenty weeks later, the paraffin-embedded uterine tissues were acquired from the mice, which were euthanized. Sections of 4-μm thick, paraffin-embedded tissue were deparaffinized with xylene and subsequently dehydrated through a series of graded ethanol baths. Expression levels of LSD1 (1:800, Cell Signaling, Danvers, MA, USA), KEAP1 (1:8000; Proteintech, Rosemont, IL, USA) and phospho-p62 (1:100; Cell Signaling, Danvers, MA, USA) in the murine uterine epithelial cells were detected using specific antibodies in an automated immunohistochemical stainer (Leica Bond Polymer Refine Detection Kit; Buffalo Grove, IL, USA) according to the manufacturer’s protocol. Hematoxylin was used for counterstaining. 

## 3. Results

### 3.1. High Glucose Induces LSD1 Expression and p62 Phosphorylation

**Figure 1:**
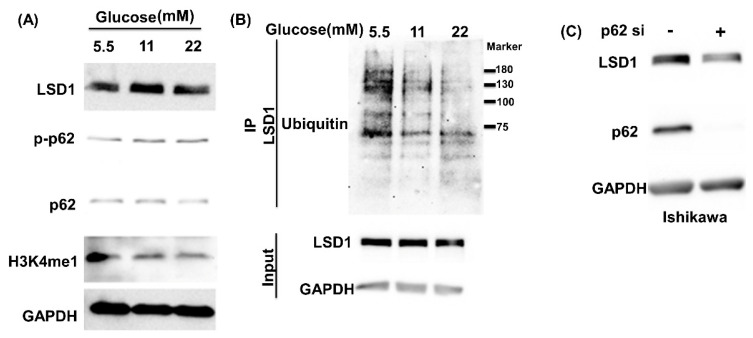

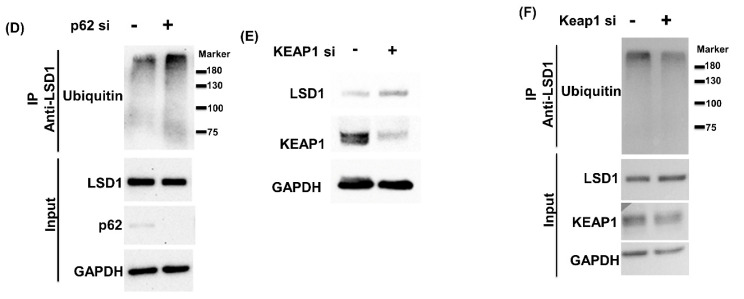
Glucose induces the p62-KEAP1 pathway to promote LSD1 expression. (**A**) Endogenous expression levels of LSD1, phospho-p62, total p62, H3K4me1 (mono-methylated histone3 Lysine 4), and GAPDH were detected in Ishikawa cells that were cultured in a 5.5, 11 or 22 mM glucose RPMI medium with 10% FBS for 48 h with specific antibodies. (**B**) Ishikawa cells were maintained in 5.5, 11 or 22 mM glucose RPMI with 10% FBS for 48 h and were then treated with MG132 for 5 h before being harvested. Subsequently, LSD1 in cell lysates was pulled down with an anti-LSD1 antibody, and ubiquitinated LSD1was identified using an antiubiquitin antibody. (**C**,**E**) Ishikawa cells were transfected with control siRNA, p62 siRNA, or KEAP1 siRNA for 72 h. Detection of endogenous LSD1, p62, KEAP1, and GAPDH was accomplished through Western blot using specific antibodies. GAPDH served as loading control. (**D**,**F**) Ischikawa cells were transfected with control, p62, or KEAP1 siRNA and harvested after 10 µM MG132 treatment for 5 h. FLAG-LSD1 was pulled down with anti-FLAG M2 antibodies, and ubiquitinated LSD1 protein was examined with anti-ubiquitin antibodies. Anti-FLAG tag (FLAG-LSD1) and GAPDH served as input controls.

We first examined the expression levels of LSD1 in primary endometrial stroma cells, which were maintained in 5.5, 11, and 22 mM glucose media for 6 days. LSD1 protein levels were elevated in high glucose media (Supplementary Figure S1). Since endometrial cancers have been divided into estrogen dependent (type I) and independent (type II). In order to fully understand the mechanisms of the induction of LSD1 by glucose, we chose two type I cell lines (HEC1B and Ishikawa cell) and one type II cell line (ARK2) to further analysis. Ishikawa, HEC1B, and ARK2 cells also had dose-dependently elevated LSD1 expression in glucose (Figure 1A; Supplementary Figure S2). Moreover, the phosphorylated p62 at serine 349 was increased, and the LSD1 substrate, methylated histone 3, was demethylated in 22 mM glucose media (Figure 1A; Supplementary Figure S2). Ubiquitinated LSD1 was higher in 5.5 mM glucose concentration media (Figure 1B). To confirm the involvement of the KEAP1–p62 pathway in the regulation of LSD1 expression, we performed siRNA knockdown of endogenous p62. LSD1 decreased in p62-suppressed cell lines (Figure 1C; Supplementary Figure S3A), and ubiquitinated LSD1 increased in the presence of p62 siRNA (Figure 1D). The knockdown of endogenous KEAP1, however, increased LSD1 expression and decreased ubiquitinated LSD1 in KEAP1-knockdown endometrial cells (Figure 1E,F; Supplementary Figure S3B). Collectively, these results indicate that glucose may promote LSD1 expression through the KEAP1–p62 pathway.

### 3.2. LSD1 Interacts with KEAP1

**Figure 2:**
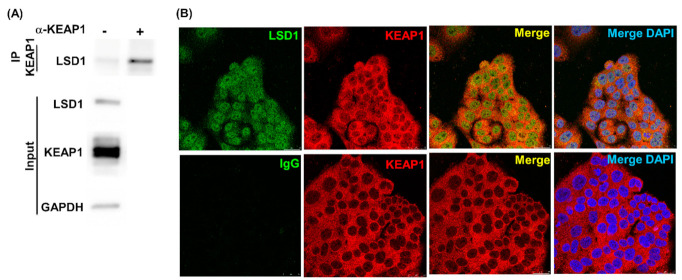

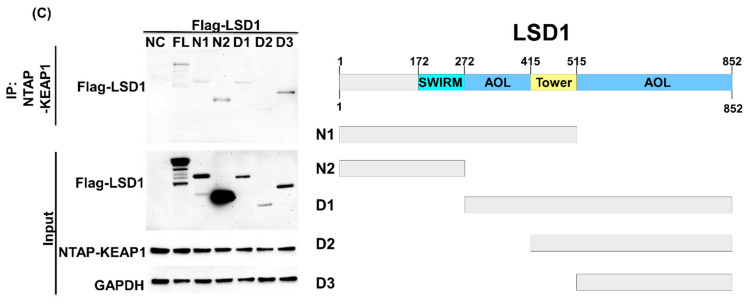

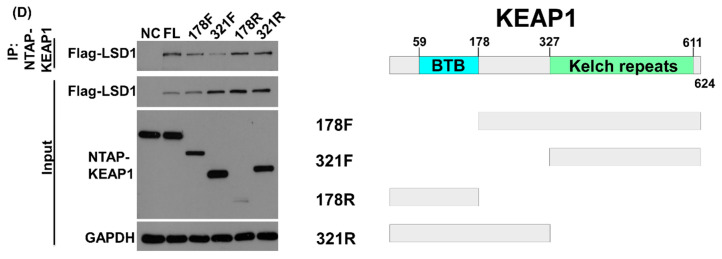
LSD1 and KEAP1 form a complex. (**A**) An endogenous KEAP1 protein complex in Ishikawa cell lysates was precipitated with KEAP1 antibodies and control IgG. KEAP1 interacting proteins, including LSD1, were identified with specific antibodies. The expression levels of LSD1, KEAP1, and GAPDH in Ishikawa cells are illustrated in the lower panel. (**B**) Colocalization of KEAP1 and LSD1 were observed through confocal microscopy. Ishikawa cells were stained with LSD1 (green signal, left panel) and KEAP1 (red signal, second one from the left panel) or control IgG (green signal, left panel) and KEAP1 (red signal, second one from the left panel). Overlapping green and red signals revealed the collocal-zation of two proteins, as denoted by the yellow signal (second one from the right panel). The right panel is overlapping green, red and blue (DAPI-nuclear staining). (**C**) Full-length KEAP1 and various truncated LSD1 (full-length, N1, N2, D1, D2, and D3; FLAG tag) were cotransfected into HEK293 cells. The truncated LSD1 proteins that interacted with KEAP1 were purified with streptavidin beads (NTAP-KEAP1), and LSD1 proteins were identified with anti-FLAG-tag antibodies. (**D**) Full-length LSD1 coprecipitated with both full-length and four truncated KEAP1 (178F, 321F, 178R, and 321R) constructs. After pull down with streptavidin beads (NTAP-KEAP1), the purified LSD1 protein was examined using anti-FLAG antibodies.

The interactions between LSD1 and p62 were reported in our previous study [33]. Here, to further examine the role played by specific domains in LSD1 and KEAP1 interactions, immunoprecipitation experiments with various truncated constructs were performed. HEK293 cells were selected as a non-malignant cell with high transfection efficiency to assay protein interactions and glucose actions in non-malignant epithelial cells. Pull-down of endogenous KEAP1 with specific antibodies identified LSD1 in the KEAP1 complex (Figure 2A). Colocalization was also confirmed using a confocal microscope (Figure 2B). We found that LSD1 associated with KEAP1 through the N-terminal (1-272, N2) and C-terminal (515-852, D3) domains (Figure 2C). Deletion of the KEAP1 N-terminal domain (178F and 321F) decreased the interaction between LSD1 and KEAP1 (Figure 2D). These data indicate that (1) the N-terminal and C-terminal domains of LSD1 are responsible for interacting with KEAP1 and (2) the N-terminal domain of KEAP1 is essential for LSD1 binding.

### 3.3. LSD1 Is Upregulated in Phospho-p62 Mimic–Expressed Cells

**Figure 3:**
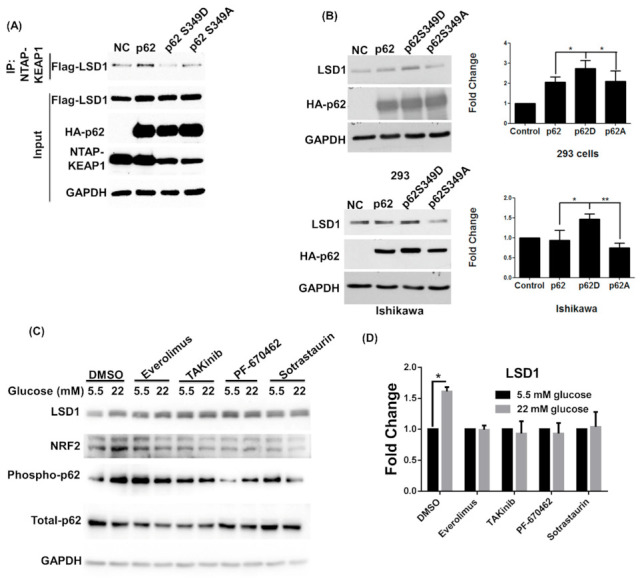

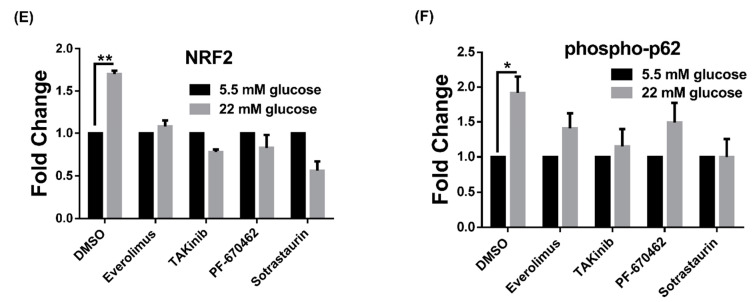
Phosphorylated p62 competes to bind with KEAP1 and induce LSD1 expression. (**A**) HEK293 cells were cotransfected with control vector (NC), wild-type p62 (p62), p62 S349D (phosphorylated p62 mimic), or p62 S349A (unphosphorylated p62) and NTAP-KEAP1 and FLAG–LSD1. Following purification with streptavidin beads (NTAP-KEAP1), interaction between KEAP1 and LSD1 was detected using anti-FLAG antibodies. (**B**) HEK293 and Ishikawa cells were transfected with control vector (NC), wild-type p62 (p62), p62 S349D (phosphorylated p62 mimic), or p62 S349A (unphosphorylated p62). The protein levels of endogenous LSD1, GAPDH, and exogenous p62 were detected through Western blot. Fold changes in LSD1 protein levels were reported in the right panel. Results are expressed as means ± standard errors of the mean from three independent experiments; * *p* < 0.05, ** *p* < 0.01. (**C**) Ishikawa cells were maintained in the indicated glucose concentrations in RPMI with 10% FBS for 72 h. Cells were treated with everolimus (mTOR inhibitor), takinib (TAK inhibitor), PF-670462 (casein kinase inhibitor), and sotrastaurin (PKC inhibitor) 12 h before harvest in the presence of proteasome inhibitor MG132 (concentration: 5 μM). Protein levels of LSD1, NRF2, phosphorylated and total p62, and GAPDH were examined through Western blot. (**D**) LSD1, (**E**) NRF2 and (**F**) phospho-p62 were quantified the changes of protein levels. Results are expressed as mean ± standard errors from three independent experiments; * *p* < 0.05, ** *p* < 0.01.

The phosphorylation of p62 on serine 349 can bind with KEAP1 and release NRF2 to avoid degradation [22]. Therefore, we also evaluated whether phosphor-p62 (S349) affects the expression level of LSD1. Of two p62 mutants, p62 S349D mimicked phosphorylated p62, and p62 S349A represented dephosphorylated p62. Compared with p62 wild type and p62 S349A, overexpressed p62 S349D decreased the interaction between LSD1 and KEAP1 (Figure 3A) and then induced LSD1 expression in nonmalignant HEK293 cells and EC cells (Figure 3B). Several kinases, including PKC [36], casein kinase 1 [37], mammalian target of rapamycin complex 1 [22], and TAK1 [38], have been reported to phosphorylate p62 at serine 349. Therefore, we used specific inhibitors to identify upstream kinases. We found that an everolimus (an mTOR inhibitor), TAKinib (a TAK inhibitor), PF-670462 (a casein kinase inhibitor), and sotrastaurin (a PKC inhibitor) can block the expression levels of LSD1, NRF2, and phosphorylated p62 (Figure 3C–F). These results indicate that p62 phosphorylation can reduce LSD1 interaction with KEAP1 and prevent the ubiquitination of LSD1. 

### 3.4. NRF2 Connects KEPA1 to LSD1

**Figure 4:**
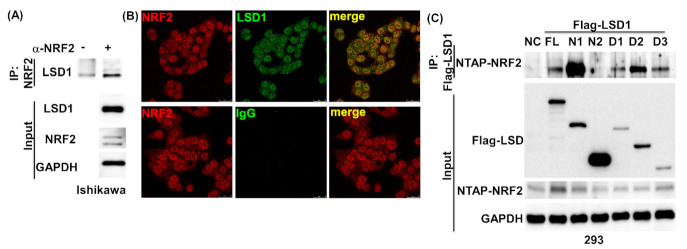

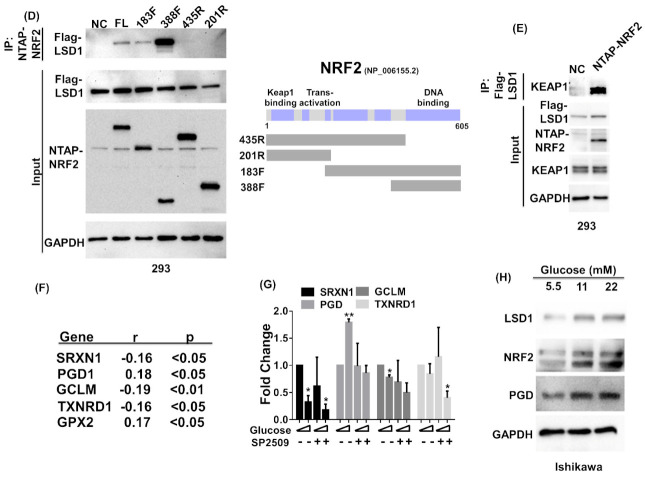
LSD1 binds with NRF2 and regulates NRF2 downstream genes expression. (**A**) Using MG132 treated (5 µM for 12 h) Ishikawa cell lysates, endogenous NRF2 complex was pulled down with anti-NRF2 or control IgG antibodies. Associated LSD1 was identified using specific antibodies. Endogenous expression of LSD1, NRF2, and GAPDH in Ishikawa cells is displayed in lower panel. (**B**) Confocal microscopy was used to examine the NRF2-LSD1 complex in Ishikawa cells. Cells were hybridized with antibodies against NRF2 (red signal, left panel), LSD1 (green signal, central upper panel), or control IgG (central bottom panel). Colocalization of NRF2 (red) and LSD1 (green) on merged images (right panel). (**C**) Full-length NRF2 and truncated LSD1 constructs (full-length, N1, N2, D1, D2, D3; FLAG tag) were cotransfected into HEK293 cells. After precipitating the complex with anti-FLAG beads, the presence of NRF2 in complex was detected using anti-CBP-tag antibodies. (**D**) Full-length FLAG-LSD1 cotransfected with truncated NRF2 (full-length, 1-435, 1-201, 183-605, 388-605; CBP tag) into HEK293 cells. Following purification with streptavidin beads (NTAP-NRF2 construct), coprecipitated LSD1 was identified using an anti-FLAG antibody. (**E**) Full-length FLAG-LSD1 was cotransfected with control vector or NTAP-NRF2 into HEK293 cells. The cells were treated with 5µM MG132 overnight before being harvested. Anti-FLAG beads were used to purify the LSD1 complex, and associated KEAP1 was detected using anti-KEAP1 specific antibodies. (**F**) The Cancer Genome Atlas uterine corpus endometrial carcinoma RNA sequence data were analyzed for correlations between NRF2 downstream target genes and LSD1 in RNA expression levels. The sign of the correlation coefficient (r) indicates whether the direction of the relationship is positive (direct) or negative (inverse). (**G**) Ishikawa cells were cultured in low-glucose (5.5 mM) or high-glucose (22 mM) RPMI with 10% FBS for 36 h. The cells were treated with vehicle control or SP2509 (LSD1 inhibitor) for 12 h before being harvested. The expression levels of target genes were quantified using real-time RT-QPCR, and then we used GAPDH genes as a loading control. Results are expressed as means ± standard errors of the mean from three independent experiments; * *p* < 0.05, ** *p* < 0.01. (**H**) Ishikawa cells were maintained in 5.5, 11, and 22 mM glucose RPMI with 10% FBS for 72 h. The expression levels of endogenous LSD1, NRF2, PGD, and GAPDH were detected through Western blot. GAPDH served as loading control.

NRF2 can be regulated by the p62–KEAP1 pathway [22]. We therefore investigated whether LSD1 expression by the p62–KEAP1 pathway depends on NRF2. After the knockdown of NRF2 by using specific antibodies, LSD1 was identified as part of the NRF2 complex (Figure 4A). This interaction was clearly confirmed in the nucleus and cytosol through confocal microscopy (Figure 4B). Using various truncated constructs in immunoprecipitation experiments helped us map the regions of interaction of these two proteins. We found that the AOL and TOWER domain (272–852) of LSD1 were responsible for its interaction with NRF2 (Figure 4C). Notably, decreased interaction between NRF2 and LSD1 was identified in 435R and 201R (Figure 4D). Moreover, overexpressed NRF2 enhanced the interaction of KEAP1 and LSD1 when treated with MG132 (Figure 4E). These results demonstrate that the AOL–TOWER domain of LSD1 and DNA-binding domain of NRF2 interact with each other and that the interaction between KEAP1 and LSD1 increases in the presence of NRF2 expression. 

### 3.5. LSD1 Participates in NRF2-Regulated Gene Expression

Because LSD1 can associate with transcription factors to regulate downstream genes expression [39], we analyzed whether LSD1 is involved in NRF2 downstream gene regulation. Several genes (*GCLC*, *GCLM*, *GSR1*, *XCT*, *GPX2*, *GSTA 1/2/3/5*, *GSTM 1/2/3*, *GSTP1*, *NQO1*, *TXN*, *TXNRD1*, *SRXN1*, *G6PD*, *PGD*, *IDH1*, *ME1*, *HMOX1*, *FTL*, and *FTH*) involved in antioxidant response have been reported as *NRF2* target genes [40], and we initially examined the expression correlations between these genes and LSD1 using The Cancer Genome Atlas EC RNA sequence dataset (*n* = 177). Five genes, namely *SRXN1*, *PGD*, *GCLM*, *TXNRD1*, and *GPX2*, exhibited significant correlations with the RNA expression of LSD1 (Figure 4F). Only the phosphogluconate dehydrogenase (*PGD*) gene was induced significantly by glucose (Figure 4G,H) and blocked in the presence of the LSD1-specific inhibitor SP2509 (Figure 4G). Therefore, LSD1 is involved in NRF2-regulated antioxidant gene expression. 

### 3.6. Cell Proliferation Induced by Glucose Is Blocked by p62 siRNA

**Figure 5:**
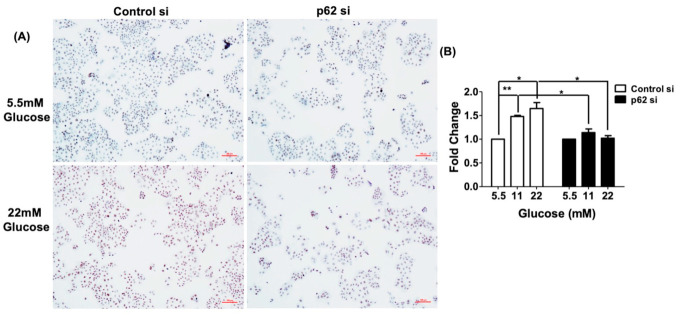

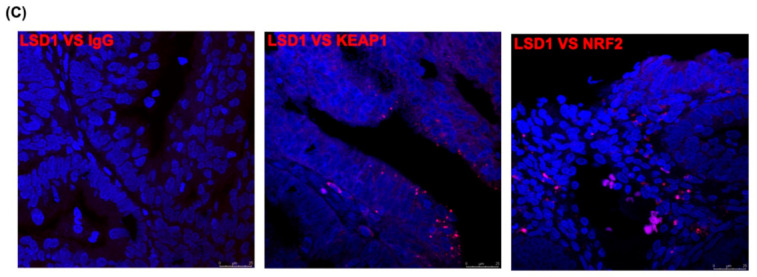

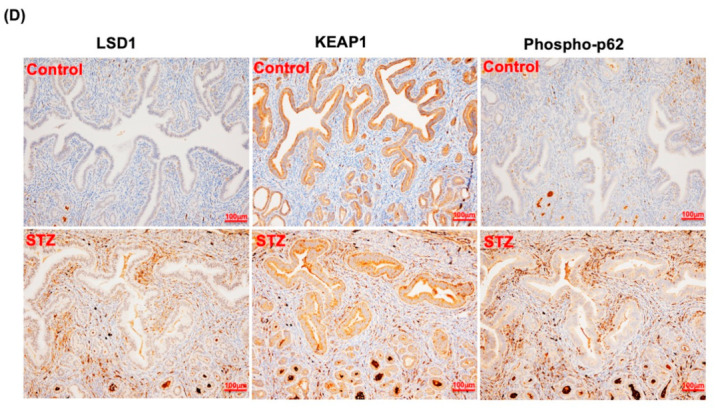
p62 is involved in regulation of cell proliferation by glucose. Cell proliferation activity was measured through (**A**) Ki-67 staining, and (**B**) BrdU assay demonstrated the differences in cell proliferation ability in the absence or presence of p62 siRNA. Ishikawa cells were transfected with control siRNA or p62 siRNA for 48 h and were subsequently maintained in indicated concentrations of glucose RPMI with 10% FBS for 24 h. Brown color cells indicated Ki67-positive proliferating cells in Ki67 staining. Results are expressed as means ± standard errors of the mean from three independent experiments; * *p* < 0.05, ** *p* < 0.01. (**C**) Proximity ligation assay confirmed interactions between LSD1, KEAP1, and NRF2 in human endometrial specimens. Tissues were hybridized with antibodies against LSD1, KEAP1, and NRF2, with IgG serving as negative control. Red dots indicate protein interactions. (**D**) Immunohistochemical staining of LSD1 (left panel), KEAP1 (middle panel), and phospho-p62 (right panel) in uterine epithelial cells of control (upper panel) or STZ-induced diabetic mice (lower panel) for 20 weeks.

Because glucose can stimulate cell proliferation in EC cells [41,42], we further investigated the role of p62 in glucose-mediated cell proliferation. We measured the Ishikawa cells’ proliferation ability through the BrdU assay and Ki67 staining in various glucose concentrations with an RPMI medium with 10% FBS in the absence or presence of p62 siRNA. Compared with control siRNA-transfected cells, p62 silenced by specific siRNA cells maintained in a 22 mM-glucose medium produced decreased expression levels of the proliferation marker Ki67 protein (Figure 5A). BrdU DNA synthesis assay also confirmed a lower proliferation capacity in the silenced p62 cells (Figure 5B). These results indicate that p62 is highly involved in glucose-mediating cell proliferation. 

### 3.7. LSD1 Interactions with KEAP1, p62, and NRF2 In Vivo

To further confirm these interactions in vivo, we used the proximity ligation assay to examine the interactions between LSD1 and the KEAP1/p62/NRF2 complex in formalin-fixed paraffin-embedded human endometrial tissue. The results demonstrate that LSD1 interacted with KEAP1, p62, and NRF2 (Figure 5C). To further confirm that glucose induces LSD1 expression, we induced diabetic mice with STZ to specifically target pancreatic beta cells and elevate the blood glucose concentration [43], and we observed the expression levels of LSD1, KEAP1, and phospho-p62 in the mice’s uterine tissue. After 20 weeks, higher LSD1 and expression was evident in the diabetic mice compared with the control mice (Figure 5D). These results reveal that high glucose induces LSD1 and phospho-p62 expression in vivo.

## 4. Discussion

**Figure 6:**
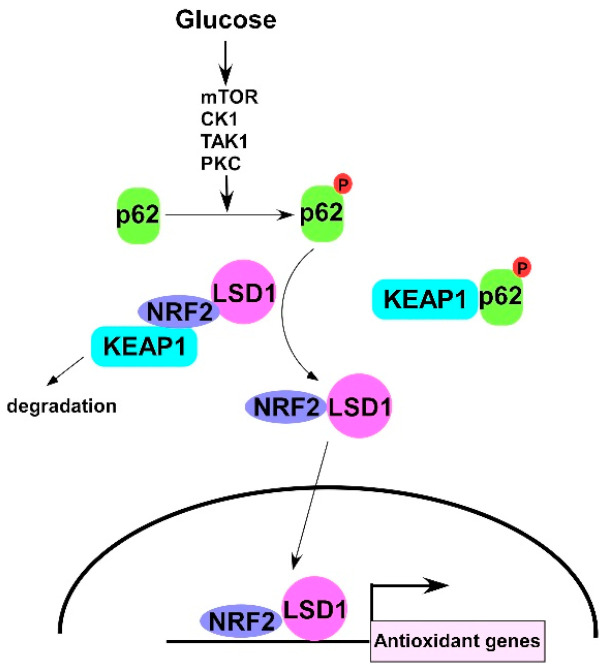
Schematic of this study. Glucose triggers p62 phosphorylation through mTOR/CK1/TAK1/PKC kinases and releases LSD1 protein from the KEAP1 complex to stabilize protein expression. Finally, the NRF2-LSD1 complex translocates to the nucleus and regulates NRF2 downstream antioxidant genes expression.

The results of our study indicate that glucose activated LSD1 induces the mTOR/CK1/TAK1/PKC signaling pathway and triggers p62 phosphorylation, which, in turn, releases the LSD1 protein from a complex formed with KEAP1. The NRF2–LSD1 complex translocates to the nucleus and activates downstream antioxidant genes (Figure 6). Our findings are the first to demonstrate that glucose concentration can regulate LSD1 protein stability through p62 phosphorylation. These observations offer more support for correlations between obesity or hyperglycemia and the incidence of EC, and they have notable therapeutic implications. Metformin can decrease blood glucose concentrations and is used to treat type 2 diabetes [44]. A study of 478,921 women with diabetes indicated that the incidence of EC considerably decreased in women who used metformin compared with those who had never used it [45]. The intake of high-sugar foods is also positively correlated with EC [46] because the expression levels of LSD1 are associated with tumorigenesis and LSD1 is considered an oncoprotein [47]. A limitation of this study is that correlation of LSD1 with the changes of reactive oxygen species (ROS) is not performed. However, our results still demonstrate that the involvement of the glucose/KEAP1/p62/NRF2/LSD1 axis may play a role in the development of EC. 

A major finding in our study is that a high concentration of glucose triggers p62 phosphorylation (Figure 1A) and decreases ubiquitinated LSD1 (Figure 3B). The expression of phosphomimic p62 (p62 S349D) reduces the interaction of LSD1 and KEAP1 (Figure 3A) and induces LSD1 expression in EC and nonmalignant cells (Figure 3B). These results indicate that glucose regulates LSD1 protein stability through the KEAP1–p62 pathway. Because LSD1 interacts with NRF2 and the LSD1–KEAP1 interaction is enhanced in the presence of NRF2 (Figure 4E). Under normal conditions, most of p62 is unphosphorylated, and the NRF2–LSD1 complex is degraded through KEAP1-mediated proteasomes to maintain the complex at a low level of expression. A high level of glucose stimulates the phosphorylation of p62, which increases the expression of the NRF2–LSD1 complex through dissociation with KEAP1. Finally, the NRF2–LSD1 complex coregulates downstream antioxidant expression in the nucleus (Figure 6). Although glucose can also glycosylate and stabilize proteins [48], LSD1 has not been reported to be a glycosylated protein, and our data clearly indicate that p62 is a major regulator in the control of LSD1 expression in the presence of high concentrations of glucose. 

p62/SQSTM1 is a stress-induced protein that interacts with various proteins to mediate multiple signaling and cellular functions, including oxidative stress response [49]. Stressors, such as starvation [50], estrogen [51], and high glucose—as determined in this study, can trigger p62 phosphorylation at serine 349, and phosphorylated p62 subsequently competitively interacts with KEAP1 and releases NRF2 from KEAP1-mediated proteasomes [49]. Our previous study reported findings concerning the interaction of p62 with LSD1 in EC and its destabilization [33]. However, we did not clarify the detailed method of how LSD1 regulates p62 protein stability. Based on this current study, we believe that LSD1 decreases p62 protein stability through KEAP1-mediated proteasomes. Because p62 competitively binds to the KEAP1 proteasome complex and releases LSD1 away from degradation, p62 may be an essential protein in the regulation of LSD1 expression on the translational level. 

Elevated blood glucose concentrations are associated with various cancers, such as liver and pancreatic cancers and EC [52,53,54]. High glucose levels can induce gene mutation [14], increase the metabolic pathway, and generate ROS and glycosylate proteins to promote tumor cell growth [4]. They also activate several oncoproteins, including JAK1/2, STAT3, and FOXO1, in endometrial cells [4]. We also observed the activation of p62/KEAP1/LSD1 signaling in the STZ mice group (Figure 5D) but identified no perceptible pathologically changes. Because LSD1 overexpression is associated with tumor development, we contend identification of this pathway will facilitate the detection of endometrial tumorigenesis at earlier stages. Our results can assist in preventing EC.

## 5. Conclusions

In summary, the results of this study indicate how glucose stabilizes LSD1 by inducing KEAP1/p62 signaling. p62 serves as a hub for LSD1 activation and cell proliferation in the presence of high concentrations of glucose. Our findings indicate how molecularly, an unhealthy diet may lead to endometrial tumorigenesis and suggest a novel therapeutic strategy involving targeting the KEAP1/p62 signaling axis. 

## Data Availability

Data is contained within the article or Supplementary Materials.

## Supplementary Materials

The following are available online at https://www.mdpi.com/article/10.3390/antiox10121898/s1, Figure S1: Glucose induced LSD1 expression in primary uterine stroma cells, Figure S2: Glucose induced p62 phosphorylation and LSD1 expression in endometrial cancer cells, Figure S3: The regulation of LSD1 by glucose was depended on KEAP1/p62 pathway, Table S1: Sequences of primers used in the study, Table S2: Materials.
