# Supplementary material for: Glucose Activates Lysine-Specific Demethylase 1 through the KEAP1/p62 Pathway
Source: Antioxidants (Basel). 2021 Nov 26;10(12):1898. doi: 10.3390/antiox10121898 (PMC8750790; doi:10.3390/antiox10121898)
Supplement: Supplementary file 1 [file antioxidants-10-01898-s001.zip › antioxidants-1464523-supplementary.pdf]

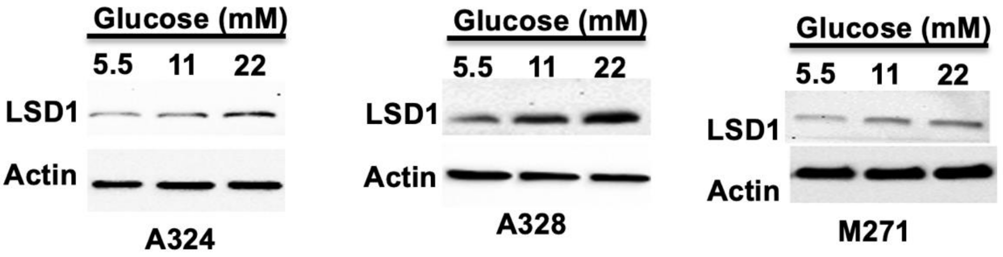

Figure S1. Glucose induced LSD1 expression in primary uterine stroma cells. Three primary endometrial stroma cells were maintained in 5.5, 11- or 22-mM glucose RPMI medium with 10% FBS for six days. Expression levels of LSD1 and GAPDH were detected with western blot. GAPDH served as loading control.

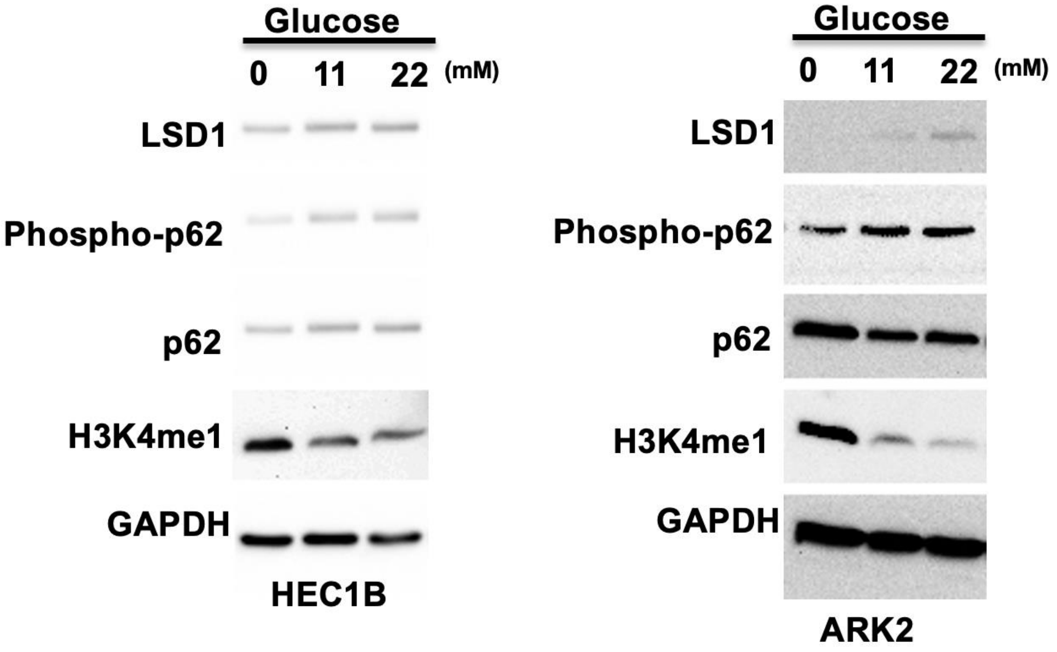

Figure S2. Glucose induced p62 phosphorylation and LSD1 expression in endometrial cancer cells. HEC1B and ARK2 cells were maintained in indicated concentration of glucose in RPMI medium with 10% FBS for 48 hours. The expression levels of LSD1, phospho-p62, total p62, monomethylated histone 3 lysine 4 (H3K4me1) and GAPDH were detected with western blot. GAPDH served as loading control.

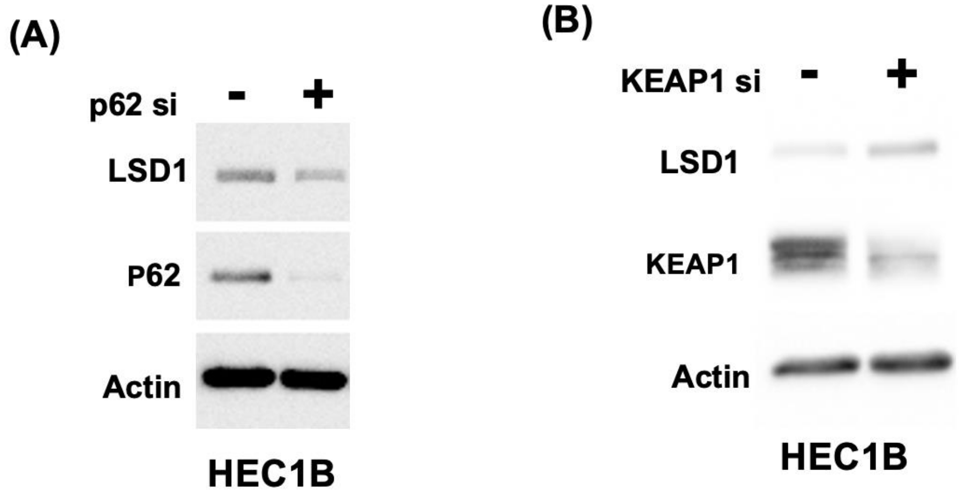

Figure S3. The regulation of LSD1 by glucose was depended on KEAP1/p62 pathway. Endometrial cancer cell-HEC1B was transfected with (**A**) p62 siRNA and (**B**) KEAP1 siRNA for 72 h. Endogenous levels of LSD1, GAPDH, p62 or KEAP1 were detected with western blot. GAPDH was as a loading control.

Table S1. Sequences of primers used in the study.

Primer	Sequence (5′—3′)
P62 S349A F	AGTGGACCCGGCTACAGGTGA
P62 S349A R	TCTTTTGAAGACAGATGGGTCCAG
P62 S349D F	AGTGGACCCGGATACAGGTGAAC
P62 S349D R	TCTTTTGAAGACAGATGGG
Keap1 3xFlag F	TAGCCCGGGCGGATCCATGCAGCCAGATCCCAGGC
Keap1 3xFlag R	ATCGATAAGCTTGATATCACAGGTACAGTTCTGCTGGTCAATC
Keap1 180 R	ATCGATAAGCTTGATATCCTGCTGCACCAGGAAGTCAC
Keap1 180 F	TAGCCCGGGCGGATCCCTGGACCCCAGCAATGCC
Keap1 327 F	TAGCCCGGGCGGATCCCCCAAGGTGGGCCGCCTG
NRF2 F	TAGCCCGGGCGGATCCATGATGGACTTGGAGCTGC
NRF2 R	CGGTATCGATAAGCTTCTAGTTTTTCTTAACATCTGGCTTC
NRF2 183 F	CTGCCCGGGCGGATCCATGATTGAGCAAGTTTGGGAGG
NRF2 388F	CTGCCCGGGCGGATCCATGAAACAGAATGGTCCTAAAACAC
NRF2 435R	CGGTATCGATAAGCTTACCAGGACTTACAGGCAATTCTTTC
NRF2 201R	CGGTATCGATAAGCTTATTAAGACACTGTAACTCAGG
GCLM QPCR-F	CCTATTGAAGATGGAGTTA
GCLM QPCR-R	CTATTTGGTTTTACCTGTG
TXNRD1 QPCR F	GAGAATATAATACGGTGATG
TXNRD1 QPCR R	AATGGCATAGATGTAAGG
SRXN1 QPCR F	AGGTGACTACTTCTACTC
SRXN1 QPCR R	CTTAGGTCTGAGAGAGTG
Primer	Sequence (5′-3′)
PGD QPCR F	CAGTTTGATGGTGATAAG
PGD QPCR R	TAGGAATACACTTCTAATGA

Table S2. Materials.

**Cell culture**	HEC1B cells were obtained from the American Type Culture Collection (Manassas, VA, USA). ARK2 cells were gifts from Dr. Alessandro D. Santin (Yale University School of Medicine, New Haven, CT, USA). The HEC1B and ARK2 cells were cultured in RPMI with 10% fetal bovine serum and 1% penicillin and streptomycin.
**Tissue specimens**	This study was reviewed and approved by the Institutional Review Board of Linkou Chang Gung Memorial Hospital (approval number: 201801952B0A3). Signed informed consent was obtained before surgery and tissue collection. Endometrial tissues were obtained from surgical specimens of fertile women with regular menstrual cycles who received laparoscopy or hysterectomy for benign gynecologic conditions.
**Isolation and maintenance of primary endometrial stroma cells**	The minced surgical endometrial tissues were digested in Hanks' Balanced Salt Solution (Invitrogen, Waltham, MA, USA) with collagenase B (15 U/mL; Roche, Basel, Switzerland), deoxyribonuclease I (150 U/mL, Roche, Basel, Switzerland), and penicillin/streptomycin at 37 °C for 60 min under agitation. Primary endometrial stroma cells were isolated through passage through 40-μm cell sieves (BD, Franklin Lakes, NJ, USA) and were cultured in DMEM/F12 with 10% fetal bovine serum[35].
